# Chronic inflammatory diseases, anti-inflammatory medications and risk of prostate cancer: a population-based case-control study

**DOI:** 10.1186/s12885-019-5846-3

**Published:** 2019-06-21

**Authors:** Kerri Beckmann, Beth Russell, Debra Josephs, Hans Garmo, Christel Haggstrom, Lars Holmberg, Pär Stattin, Mieke Van Hemelrijck, Jan Adolfsson

**Affiliations:** 1 UniSA Cancer Research Institute, University of South Australia, Adelaide, Australia; 2 School of Cancer and Pharmaceutical Studies, Translational Oncology & Urology Research (TOUR), King’s College London, London, UK; 3 Regional Cancer Centre Uppsala, Uppsala University Hospital, Uppsala, Sweden; 4 Department of Biobank Research, Umea University, Umea, Sweden; 5 Department of Surgical Sciences, Uppsala University Hospital, Uppsala, Sweden; 6 Unit of Epidemiology, Institute of Environmental Medicine, Karolinska Institutet, Stockholm, Sweden; 7 CLINTEC-department, Karolinska Institutet, Stockholm, Sweden

**Keywords:** Prostate cancer, Chronic inflammatory disease, Autoimmune disease, Anti-inflammatory medication

## Abstract

**Background:**

Whether chronic inflammation increases prostate cancer risk remains unclear. This study investigated whether chronic inflammatory diseases (CID) or anti-inflammatory medication use (AIM) were associated with prostate cancer risk.

**Methods:**

Fifty-five thousand nine hundred thirty-seven cases (all prostate cancer, 2007–2012) and 279,618 age-matched controls were selected from the Prostate Cancer Database Sweden. CIDs and AIMs was determined from national patient and drug registers. Associations were investigated using conditional logistic regression, including for disease/drug subtypes and exposure length/dose.

**Results:**

Men with a history of any CID had slightly increased risk of any prostate cancer diagnosis (OR: 1.08; 95%CI: 1.04–1.12) but not ‘unfavourable’ (high-risk or advanced) prostate cancer. Generally, risk of prostate cancer was highest for shorter exposure times. However, a positive association was observed for asthma > 5 years before prostate cancer diagnosis (OR: 1.21; 95%CI: 1.05–1.40). Risk of prostate cancer was increased with prior use of any AIMs (OR: 1.26; 95%CI: 1.24–1.29). A positive trend with increasing cumulative dose was only observed for inhaled glucocorticoids (*p* < 0.011).

**Conclusion:**

Detection bias most likely explains the elevated risk of prostate cancer with prior history of CIDs or use of AIMs, given the higher risk immediately after first CID event and lack of dose response. However, findings for length of time with asthma and dose of inhaled glucocorticoids suggest that asthma may increase risk of prostate cancer through other pathways.

**Electronic supplementary material:**

The online version of this article (10.1186/s12885-019-5846-3) contains supplementary material, which is available to authorized users.

## Background

There is convincing evidence that some specific cancers are associated with chronic inflammatory diseases (CIDs). Examples include inflammatory bowel disease and colon cancer, *Helicobacter Pylori* infection and gastric cancer, hepatitis B and C infections and hepatocellular carcinoma [1]. Evidence for chronic inflammation as a risk factor for prostate cancer is less clear. Several different lines of evidence suggest that inflammation plays a role in prostate cancer development. For example, a number of putative risk factors for prostate cancer induce prostate inflammation, including heterocyclic amines in highly cooked meat, prostatitis and sexually transmitted diseases (STDs) [2–5]. In addition to localised inflammation, there is evidence suggesting systemic inflammation may also increase prostate cancer risk. High levels of circulating C-reactive protein [6], a marker of inflammation, and increased Immunoglobulin (Ig) E levels [7], a marker of atopy, are associated with increased risk of prostate cancer. Increased risk of prostate cancer has been reported among men with asthma or other atopic conditions [7, 8] and among men with autoimmune diseases [9–12], though contradictory findings have also been reported [13–17]. On the other hand, reduced risk of prostate cancer has been reported with exposure to some anti-inflammatory medications (AIMs) used to manage CIDs, though again the evidence is mixed [18–21].

However, previous evidence of positive associations between CIDs/AIMs and prostate cancer may be affected by detection bias, resulting from frequent health service encounters or investigations leading to detection of prostate cancer, especially for men with localised inflammation arising from prostatitis or STDs [22].

The aim of this study was to investigate the link between chronic inflammation and risk of prostate cancer by examining the association between 1) history of CIDs, and 2) use of AIMs, and prostate cancer incidence, using population-wide registry-based data. Our a priori hypotheses were that risk of prostate cancer would be increased among men with CIDs due to persistent or recurring exposure to inflammatory mediators, while risk would be reduced among men who used AIMs, due to anti-inflammatory properties of these drugs.

## Methods

### Data source and variables

We used data from Prostate Cancer Database Sweden (PCBaSe), a population-wide database linkage between the National Prostate Cancer Registry (NPCR), the National Patient Register (NPR), the National Prescribed Drug Register (from Jan 1, 2006) and the Swedish longitudinal integration database for health insurance and labour market studies (LISA), through Swedish personal identification numbers [23]. In addition to men diagnosed with prostate cancer, PCBaSe also includes disease-free population controls, selected using an incident density sampling approach [23]. The NPCR has near complete coverage (98%) of prostate cancer diagnoses across Sweden since 1988. Information collected in NPCR includes date of diagnosis, age, tumour stage and grade, levels of prostate specific antigen (PSA) at diagnosis, and primary treatment.

We used a case-control design to study the association between CIDs/AIMs and risk of prostate cancer diagnosis. The main outcome of this study was a diagnosis of prostate cancer, recorded in NPCR. All men diagnosed with prostate cancer between Jan 1, 2007 and Dec 31, 2012 were selected as cases. Five population controls, free of prostate cancer at the time of diagnosis of the corresponding index case, matched on year of birth and area of residence, were selected for each case.

The two broad exposures of interest were 1) prior history of CIDs and 2) exposure to AIMs. For the CIDs component, the analytic study population was restricted to men diagnosed from 2007 to 2009 plus controls, due to incomplete data on rare conditions from 2010 onwards. Prior history of CIDs was derived from International classification of diseases, 10th edition (ICD-10) codes recorded in the NPR for any single hospital inpatient or outpatient episode from 1998 onwards. CIDs were classified into the following subcategories: chronic inflammatory and infectious diseases; allergies; and autoimmune diseases (further sub-grouped as auto-antibody negative; auto-antibody positive – systemic; and auto-antibody positive - organ specific diseases). Type 1 diabetes mellitus (T1DM) was excluded from the organ specific autoimmune category given likely misclassification of type 2 diabetes mellitus. Likewise, prostatitis was excluded from the chronic inflammatory diseases category due to likely detection bias from more intensive investigation of the prostate. Both were analysed separately. The full list of included diseases and respective ICD-10 codes is provided in the Additional file 2.

For analysis of AIMs, the whole study population was included. Exposure to selected AIMs was determined through linkage to Sweden’s National Prescribed Drug register, based on Anatomical Therapeutic Chemical (ATC) codes for drugs commonly prescribed for the management of CIDs: Non-aspirin non-steroidal anti-inflammatory drugs (NSAID) (M01A), systemic glucocorticoids (H02AB), inhaled glucocorticoids (R03BA), non-steroidal asthma medications (R03AC, R03CC, R03AK, R03BB, R03DC), immuno-suppressants (L04A), and anti-gout medications (M04A). One previous prescription was considered to be sufficient to be classified as exposed. Data were extracted on class of drug, date of first prescription and cumulative dose before diagnosis. These data were only available from July 1, 2005, onwards. For both CIDs and AIMs, length of exposure and cumulative dose were determined for similar periods for cases and controls, with date of diagnosis of the case being used as the reference date for selected controls.

Charlson comorbidity index [24] (CCI) was derived from data hospital admission records prior to diagnosis of prostate cancer. Marital status and education level were extracted from LISA, while data on prostate cancer risk categories (grade, clinical stage, and prostate serum antigen (PSA) level at diagnosis) were extracted from NPCR. To determine whether CIDs or AIMs were associated with disease severity at diagnosis we classified men with Prostate cancer according to risk categories. The ‘favourable risk prostate cancer’ group included low and intermediate risk categories (Gleason Score < =7, clinical stage T1–2, PSA ≤ 20, N0/X and M0/X]), while the ‘unfavourable risk’ prostate cancer group included high-risk, regionally-advanced and metastatic disease (Gleason score = 8–10, PSA > 20, N1 or M1). These terms are used below to refer to different risk groupings.

### Statistical analysis

Associations between CIDs/AIMs and prostate cancer risk were examined using multivariable conditional logistic regression. Separate analyses were undertaken for any prior CIDs and any AIMs exposure, as well as each CID and AIM subtype. In all cases the reference group were all men not previously exposed to the specific condition or medication of interest. To assess the potential for detection bias, we examined length of time since first recorded diagnosis of CID (< 12, 12- < 36, 36- < 60,  60+ months) and total cumulative dose for subtypes of AIMs (< 50, 50- < 200, 200- < 500, 500+ daily dose equivalents) prior to prostate cancer diagnosis. Models were adjusted for education level (low< 10 years, middle 10–12 years, high>12 years); marital status (married, not married), and comorbidity (CCI =0, 1, 2, 3+). Wald tests were used to test trends according to categories of exposure for CIDs and cumulative daily dose for AIMs. Separate subgroup analyses were also undertaken restricted to ‘favourable’ and ‘unfavourable’ risk groups and their respective controls.

We also investigated interaction between history of CIDs and major classes of anti-inflammatory medications. For these analyses, interaction terms for CIDs and AIMs subtype were included in regression models for overall risk of prostate cancer, along with other covariates. Differences in log likelihood between models with and without interaction terms were used to determine any statistically significant effect modification in men with CID history.

Finally we undertook a series of sensitivity analyse to examine the robustness of our findings with respect to AIMs and prostate cancer risk. These included 1) altering our definition of AIMs exposure to 2 or more prior prescriptions, 2) restricting the cohort to men diagnosed with prostate cancer from 2010 to 2012 to allow for longer time for AIMs exposure given prescription data were only available from 2006, and 3) excluding cases whose prostate cancer diagnosis was within 12 months of their first prescription.

All statistical analyses were undertaken using Stata v 14.0 (Stata Corporation, College Station, Texas USA). This study was approved by the Research Ethics Board, Umeå University, Sweden.

## Results

The study population included 55,937 men with prostate cancer diagnosed between 2007 and 2012 and 279,618 controls. Among cases, 32,193 (57%) had favourable risk prostate cancer, 22,275 (43%) had unfavourable risk prostate cancer, and 1469 (3%) had unknown risk category. Table 1 presents demographic characteristics of the study population. Mean age at diagnosis and selection as controls was 69 years (standard deviation = 9). The proportion of men with a prior record of CIDs was similar between cases and controls (20% vs 19%). The proportion of men previously prescribed AIMs (all medications combined) was slightly higher for cases than controls (56% vs 50%).

**Table 1:** Characteristics of the study population

Cohort Characteristics	Cases	Controls
	*n* = 55,937	*n* = 279,618
Age at diagnosis (years)	No. (%)	No. (%)
<50	526 (1)	2656 (1)
50–59	6716 (12)	33,583 (12)
60–69	22,532 (40)	112,544 (40)
70–79	17,582 (31)	87,963 (31)
80+	8581 (15)	42,872 (15)
Marital status		
Single/divorced/widowed	18,753 (34)	103,830 (37)
Married	37,184 (66)	175,788 (63)
Education level		
<9 yrs	20,447 (37)	107,490 (38)
10-12 yrs	21,532 (38)	106,261 (38)
>12 yrs	13,532 (24)	61,699 (22)
missing	447 (1)	4168 (1)
Charlson Comorbidity Index		
0	15,268 (66)	73,485 (64)
1	3722 (17)	19,636 (17)
2	2369 (10)	11,618 (10)
3+	1824 (7)	11,169 (9)
Period of diagnosis/inclusion		
2007–2009	27,874 (50)	139,311 (50)
2010–2012	28,963 (50)	140,307 (50)

### Chronic inflammatory conditions and prostate cancer risk

Adjusted odds ratios (ORs) for associations between history of CIDs and risk of prostate cancer are shown in Table 2**.** Results indicate a modest positive association between history of any CIDs and risk of prostate cancer (OR: 1.08; 95% CI 1.04–1.12). Positive associations were also observed for two disease sub-categories: chronic inflammatory/infectious diseases (OR: 1.07: 95%CI: 1.03–1.12) and allergies (OR: 1.10; 95%CI 1.03–1.18) and specifically for asthma within the allergies category (OR: 1.13, 95%CI 1.02–1.25). No association was observed for autoimmune diseases, including by antibody status or whether systemic or organ confined. We found moderately decreased risk of prostate cancer among men with T1DM (OR: 0.71; 95%CI: 0.65–0.78) and elevated risk among men with chronic prostatitis (OR: 1.82; 95%CI: 1.53–2.16).

**Table 2:** Prior history of chronic inflammatory disease (CID) and odds of prostate cancer, overall and by disease category^a^

Prior record of chronic inflammatory disease^b^	Controls *n* = *139,311*	Cases *n* = 27,874	Overall PCa	Favourable risk PCa	Unfavourable risk PCa
	*No. (%)*	*No. (%)*	*OR* ^*c*^ *(95% CI)*	*OR* ^*c*^ *(95% CI)*	*OR* ^*c*^ *(95% CI)*
Any chronic inflammatory /autoimmune disease (excl. Prostatitis and T1DM)	26,272 (19)	5460 (20)	1.08 (1.04–1.12)	1.13 (1.08–1.19)	1.02 (0.96–1.07)
Chronic inflammatory / infectious diseases (excl. Prostatitis)	17,027 (12)	3520 (13)	1.07 (1.03–1.12)	1.10 (1.04–1.16)	1.05 (0.99–1.11)
Chronic prostatitis	492 (0.4)	180 (0.7)	1.82 (1.53–2.16)	2.03 (1.63–2.52)	1.56 (1.18–2.07)
Allergies (including Asthma)	5064 (4)	1084 (4)	1.10 (1.03–1.18)	1.22 (1.12–1.33)	0.97 (0.87–1.07)
Asthma alone	2116 (2)	456 (2)	1.13 (1.02–1.25)	1.22 (1.07–1.41)	1.03 (0.88–1.20)
Autoimmune diseases (any excluding T1DM):	7692 (5)	1542 (6)	1.03 (0.97–1.09)	1.07 (0.99–1.15)	0.98 (0.90–1.07)
Autoantibody negative	5349 (4)	1103 (4)	1.05 (0.98–1.12)	1.08 (0.99–1.18)	1.00 (0.90–1.10)
Autoantibody positive - systemic	1464 (1)	296 (1)	1.07 (0.94–1.21)	1.07 (0.90–1.28)	1.06 (0.89 = 1.27)
Autoantibody positive - organ specific	5221 (4)	774 (3)	0.77 (0.71–0.83)	0.74 (0.67–0.83)	0.82 (0.73–0.91)
Autoantibody positive - organ specific (excl. T1DM)	1152 (1)	220 (1)	0.98 (0.84–1.12)	1.08 (0.89–1.31)	0.85 (0.68–1.06)
T1DM	4136 (3)	506 (2)	0.71 (0.65–0.78)	0.64 (0.56–0.73)	0.80 (0.71–0.91)

Aberrations: *CI* confidence interval, *OR* Odds Ratio, *PCa* prostate cancer, *T1DM* Type 1 diabetes mellitus

^a^Favourable risk PCa: low and intermediate risk (Gleason Score ≤ 7, T1-T2, PSA ≤ 20 ng/mL, N0/X, & M0/X), Unfavourable risk PCa: (Gleason score 8–10, or PSA > 20 ng/ml, or N1, or M1)

^b^Derived from ICD-10 diagnosis codes recorded in hospital admission or outpatient records since 1997. Reference category = no record of inpatient or outpatient hospital episode

^c^Adjusted OR from conditional logistic regression with adjustment for civil status, education level and Charlson Comorbidity Index. Analyses stratified low/intermediate risk and advanced disease at diagnosis along with respective matched controls. Analysis undertaken on subset diagnosed 2007–09, with complete hospital admission data from Jan 1, 1997 – Dec 31, 2009

Associations between CIDs and prostate cancer risk groups are also shown in Table 2. For all CIDs combined, and for the inflammatory/infectious diseases and allergies subcategories, risk was only increased for favourable risk prostate cancer. Only history of chronic prostatitis was associated with both favourable (OR: 2.03; 95%CI: 1.63–2.52) and unfavourable risk prostate cancer (OR: 1.56; 95%CI: 1.18–2.07), with the former having the stronger association. The negative association observed for T1DM was evident for both favourable (OR: 0.64; 95%CI: 0.56–0.80) and unfavourable risk prostate cancer (OR: 0.80; 95%CI: 0.71–0.91), with a stronger association for favourable risk prostate cancer.

Table 3 shows the adjusted ORs for CIDs and risk of prostate cancer for time since first recorded hospital episode. There were no consistent trends with length of time since first diagnosis for any CIDs or specific subtypes. For prostatitis, there was a significant decreasing trend in prostate cancer risk with increasing length of exposure (*p* = 0.004). For the allergies subcategory, ORs were highest among men with their first hospital episode within 12 months of diagnosis (OR: 1.25; 95%CI: 1.00–1.56), but were also elevated for intervals of more than 60 months (OR: 1.19; 95%CI: 1.05–1.28). The same pattern, though more pronounced, was seen for asthma alone.

**Table 3:** Time interval from first recorded episode for specific inflammatory disease groups and odds of prostate cancer diagnosis

Time from first hospital episode to diagnosis/inclusion date	<12 months	12- < 36 months	≥ 36 months	> 60 months	
	OR^a^ (95% CI)	OR (95% CI)	OR (95% CI)	OR (95% CI)	p-trend^b^
DISEASE GROUPS *(reference: no recorded episode)*					
Any chronic inflammatory /autoimmune disease (excl. Prostatitis and T1DM)	1.01 (0.90–1.14)	1.09 (1.02–1.16)	1.04 (0.98–1.11)	1.10 (1.05–1.15)	0.371
Chronic inflammatory/infectious diseases (excl. prostatitis)	0.93 (0.80–1.07)	1.11 (1.03–1.20)	1.07 (1.03–1.12)	1.10 (1.04–1.16)	0.390
Chronic Prostatitis	3.93 (2.15–7.19)	2.29 (1.66–3.16)	1.52 (1.23–1.89)	1.53 (1.15–2.03)	0.004
Allergies (including asthma)	1.25 (1.00–1.56)	1.01 (0.88–1.15)	1.04 (0.91–1.19)	1.19 (1.05–1.28)	0.480
Asthma alone	1.63 (1.14–2.31)	0.94 (0.74–1.18)	0.99 (0.79–1.23)	1.21 (1.05–1.40)	0.700
Auto-immune diseases (excluding T1DM)	1.05 (0.84–1.32)	0.96 (0.85–1.09)	1.03 (0.91–1.16)	1.05 (0.97–1.13)	0.425
Antibody negative	1.13 (0.87–1.46)	0.98 (0.85–1.13)	1.12 (0.98–1.28)	1.03 (0.94–1.14)	0.981
Antibody positive - systemic	0.92 (0.53–1.59)	1.01 (0.76–1.34))	0.99 (0.74–1.33)	1.13 (0.96–1.33)	0.303
Antibody positive - organ confined (excl. T1DM)	0.90 (0.47–1.69)	1.01 (0.74–1.37)	0.70 (0.48–1.03)	1.06 (0.87–1.27)	0.474
T1DM only	0.97 (0.68–1.40)	0.66 (0.53–0.81)	0.71 (0.64–0.79)	0.71 (0.63–0.81)	0.673

Aberrations: *CI* confidence interval, *OR* Odds Ratio, *PCa* prostate cancer, *T1DM* Type 1 diabetes mellitus

^a^Adjusted OR from conditional logistic regression with adjustment for marital status, education level and Charlson Comorbidity Index. Analysis undertaken on subset diagnosed 2007–09, with complete hospital admission data from Jan 1, 1997 – Dec 31, 2009

^b^*P*-value for trend across time categories (Wald statistic)

### Anti-inflammatory medications and prostate cancer risk

A moderate positive association was observed for exposure to any of the listed AIMs (OR: 1.26; 95%CI: 1.24–1.29) Table 4. Increased risk was observed for separate classes of AIMs including non-aspirin-NSAIDs (OR: 1.25; 95%CI: 1.23–1.27); systemic glucocorticoids (OR: 1.14; 95%CI: 1.11–1.17), inhaled glucocorticoids (OR: 1.12; 95%CI: 1.08–1.17) and other non-steroidal asthma medications (OR: 1.11; 95%CI: 1.07–1.14). Of the non-aspirin-NSAIDs, risk of prostate cancer were elevated for acetic acid derivatives (OR: 1.24; 95%CI: 1.21–1.26), propionic acid derivatives (OR: 1.15; 95%CI: 1.12–1.18) and Cox2 inhibitors (OR: 1.18; 95%CI: 1.12–1.21) and both short acting (OR: 1.09; 95%CI: 1.03–1.16) and long acting beta agonist (OR: 1.12; 95%CI: 1.08–1.16) non-steroidal asthma medications. Sensitivity analyses with a stricter definition of exposure (≥2 prescriptions), with exclusion of cases diagnosed within 12 months of their first prescription and for the diagnosis period 2010–12 did not alter the strength or direction of association for any AIMs subtypes and overall prostate cancer risk (Additional file 1: Table S1).

**Table 4:** Prior exposure to anti-inflammatory medications and the odds of prostate cancer, overall and by disease category^a^

Exposure anti-inflammatory medications^b^	Controls *n* = 279,618	Cases *n* = 55,937	Overall PCa	Favourable risk PCa	Unfavourable risk PCa
	No. %	No. %	OR^c^ (95% CI)	OR^c^ (95% CI)	OR^c^ (95% CI)
Any Anti-inflammatory medication	141,172 (50)	31,330 (56)	1.26 (1.24–1.29)	1.34 (1.31–1.38)	1.17 (1.13–1.20)
Any Non-Aspirin-NSAIDs	112,742 (40)	25,591 (46)	1.25 (1.23–1.27)	1.33 (1.30–1.36)	1.15 (1.12–1.19)
Acetic acid derivatives	79,279 (28)	18,332 (33)	1.24 (1.21–1.26)	1.29 (1.26–1.33)	1.16 (1.12–1.20)
Propionic acid derivatives	43,766 (16)	9817 (18)	1.15 (1.12–1.18)	1.22 (1.18–1.26)	1.07 (1.03–1.11)
Cox inhibitors	7741 (3)	1828 (3)	1.18 (1.12–1.25)	1.24 (1.16–132)	1.10 (1.01–1.20)
Oxicams	2085 (1)	456 (1)	1.09 (0.98–1.21)	1.24 (1.09–1.41)	0.87 (0.73–1.04)
Other non-Aspirin NSAIDs	15,093 (5)	3334 (6)	1.10 (1.06–1.14)	1.25 (1.19–1.32)	0.92 (0.87–0.98)
Any non-topical glucocorticoid	43,052 (15)	9500 (17)	1.14 (1.11–1.17)	1.19 (1.15–1.23)	1.08 (1.04–1.12)
Systemic glucocorticoids	33,616 (12)	7449 (13)	1.14 (1.11–1.18)	1.20 (1.16–1.25)	1.08 (1.03–1.12)
Inhaled glucocorticoids	15,111 (5)	3343 (6)	1.12 (1.08–1.17)	1.15 (1.09–1.21)	1.09 (1.03–1.15)
Any (nonsteroidal) asthma medications	23,205 (8)	4948 (9)	1.11 (1.07–1.14)	1.12 (1.07–1.17)	1.09 (1.03–1.14)
Short term b-agonists	6171 (2)	1302 (2)	1.09 (1.03–1.16)	1.10 (1.02–1.20)	1.07 (0.98–1.17)
Long term b-agonists	17,840 (6)	3850 (7)	1.12 (1.08–1.16)	1.11 (1.06–1.17)	1.12 (1.06–1.18)
LAMA	7202 (3)	1441 (3)	1.06 (1.00–1.12)	1.06 (0.98–1.15)	1.05 (0.97–1.14)
LTRAs	1579 (1)	340 (1)	1.08 (0.96–1.21)	1.10 (0.95–1.28)	1.05 (0.87–1.27)
Anti-Gout medications	12,910 (5)	2568 (5)	1.03 (0.98–1.07)	1.00 (0.94–1.07)	1.05 (0.99–1.12)
Immuno-suppressants	4281 (2)	812 (1)	0.98 (0.91–1.06)	0.98 (0.88–1.08)	1.00 (0.89–1.13)

*CI* confidence interval, *LAMA* long acting muscarinic antagonists, *LTRAs* leukotriene receptor antagonists, *NSAIDs* non-steroidal anti-inflammatory drugs, OR: odds ratio

^a^Favourable risk PCa: low and intermediate risk (Gleason Score ≤ 7, T1-T2, PSA ≤ 20 ng/mL, N0/X, & M0/X), Unfavourable risk PCa: (Gleason score 8–10, or PSA > 20 ng/ml, or N1, or M1)

^b^Any exposure to anti-inflammatory medications since July 2005, derived from the National Prescribed Drug Register based on anatomical therapeutic chemical (ATC) codes

^c^Adjusted OR from conditional logistic regression with adjustment for marital status, education level and Charlson comorbidity index. Analysis undertaken in the full dataset (2007–2012)

With respect to disease severity, exposure to any AIMs was positively associated with both favourable (OR: 1.34; 95%CI: 1.31–1.38) and unfavourable risk prostate cancer (OR: 1.17; 95%CI: 1.13–1.20) but associations were stronger for the favourable risk group. Similar patterns was observed for most AIMs subtypes.

As shown in Table 5, significant trends indicating stronger associations with increasing cumulative dose were seen for inhaled glucocorticoids (*p* = 0.011) and immuno-suppressants (*p* = 0.001). However, for immune-suppressants, lower cumulative doses (<=200 daily dose equivalents) were associated with reduced risk of prostate cancer diagnosis, with no association at higher doses (> 200 daily dose equivalents). For acetic acid derivatives there was a significant trend toward decreasing association with increasing cumulative dose (*p* = 0.002). There were no significant trends for other classes of non-aspirin-NSAIDs.

**Table 5:** Cumulative dose for selected classes of anti-inflammatory medications and risk of prostate cancer

Cumulative dose of anti-inflammatory medication ^a^	<=50 dd	51–200 dd	201–500 dd	> 500 dd	
	OR^b^ (95% CI)	OR^b^ (95% CI)	OR^b^ (95% CI)	OR^b^ (95% CI)	p-trend^c^
Any na-NSAID (*reference: no prescriptions*)	1.20 (1.18–1.23)	1.34 (1.30–1.37)	1.29 (1.24–1.35)	1.19 (1.14–1.24)	0.078
Acetic acid derivatives	1.20 (1.17–1.22)	1.33 (1.29–1.38)	1.27 (1.20–1.36)	1.19 (1.09–1.30)	0.002
Propionic acid derivatives	1.15 (1.11–1.18)	1.20 (1.15–1.26)	1.14 (1.06–1.22)	1.06 (0.99–1.14)	0.226
Cox inhibitors	1.20 (1.12–1.29)	1.20 (1.09–1.32)	1.24 (1.04–1.47)	0.94 (0.76–1.15)	0.128
Systemic glucocorticoids	1.12 (1.08–1.17)	1.17 (1.11–1.23)	1.24 (1.15–1.32)	1.08 (1.01–1.16)	0.697
Inhaled glucocorticoids	1.03 (0.95–1.12)	1.09 (1.02–1.17)	1.23 (1.13–1.34)	1.16 (1.08–1.24)	0.011
Non-steroidal asthma medications	1.06 (0.99–1.14)	1.06 (0.99–1.14)	1.17 (1.09–1.26)	1.12 (1.07–1.18)	0.098
Immuno-suppressants	0.58 (0.38–0.89)	0.75 (0.60–0.92)	1.02 (0.86–1.21)	1.06 (0.96–1.17)	0.001

*CI* confidence interval, *dd* daily dose equivalents, *NSAIDs* non-steroidal anti-inflammatory drugs, *OR* odds ratio

^a^Cumulative daily dose equivalents of specific anti-inflammatory medications since July 2005, derived from the National Prescribed Drug Register based on anatomical therapeutic chemical (ATC) codes

^b^Adjusted odds ratios derived from conditional logistic regression with adjustment for civil status, education level and Charlson comorbidity index. Analysis undertaken in the full dataset (2007–2012)

^c^*P*-value for trend across cumulative dose categories (Wald statistic)

With respect to interaction between CIDs and AIMs, we observed a slight decrease in the strength of association between prior non-aspirin-NSAID use and overall prostate cancer risk among men with a history of CIDs (*P* < 0.04). Similar interactions were observed for acetic acid derivative (*p* = 0.007) and for systemic glucocorticoid exposure (*p* = 0.001) but again the effects was only slightly weaker in men with history of CIDs (Additional file 1: Table S2).

## Discussion

This comprehensive, population-wide observational study found no convincing evidence of increased risk of prostate cancer among men with a history of any CIDs. While the odds ratio for any prior CID was slightly elevated (OR = 1.08), this only applied to favourable risk disease, with no elevated risk for less favourable risk prostate cancer. For specific CIDs, our results suggest increased risk of prostate cancer for chronic prostatitis and for allergies, but decreased risk among those hospitalised with T1DM. Our analyses of AIMs revealed moderately increased risk of prostate cancer with any use of AIMs, and specifically for non-aspirin-NSAID, glucocorticoids and other non-steroidal asthma medications.

Detection bias most likely explains the association between prostate cancer and history of any CID or use of AIMs, given more frequent contact with the healthcare system would increase the opportunity for prostate cancer detection through blood tests which include PSA measurement. As in most other countries, routine PSA screening is not recommended in Sweden [25]. Nevertheless, opportunistic screening occurs frequently in Sweden, with a 5-year prevalence estimated at 60% in 2010 [26, 27]. Physicians attitudes and recommendations influence uptake of PSA testing [28, 29] and PSA testing frequency appears to be increased among those in regular contact with the healthcare system [30].

While several previous studies have reported associations between prostatitis and prostate cancer, debate is ongoing regarding whether the association is causal –i.e. via inflammatory pathways – or due to detection bias given more prostate-specific examinations [2]. Our results support the role of detection bias, since risk was highest for chronic prostatitis admissions within 12 months of diagnosis. However, given the elevated odds for chronic prostatitis > 60 months prior to prostate cancer diagnosis, it is possible that other pathways may be involved, though this is difficult to disentangle from ongoing prostatic investigations.

Previous studies have consistently reported lower risk of prostate cancer in men with diabetes [31–34]. Proposed explanations include decreased hormone levels which may slow prostate carcinogenesis, potential effects of diabetes or anti-diabetic medications on serum PSA levels or prostate tissue pathology which lead to delays in diagnosis, and reduced screening/health seeking behaviour among diabetic men which may reduce opportunities for diagnosis [34]. Though most of this evidence relates to type-2 diabetes mellitus, a multinational study using diabetes registry data has reported lower risk of prostate cancer among men with T1DM [35]. While our findings also suggest decreased risk in men with T1DM, concerns remain about the accuracy of hospital records for type of diabetes, given the higher than expected prevalence of T1DM in our cohort.

With respect to allergies/asthma, we also observed higher risk for first hospitalisation within 12 months, which indicates detection bias may play a role. However, risk was also significantly elevated for first hospitalisation > 5 years earlier suggesting the association may also involve pathways other than detection bias. Increased risk of prostate cancer has been reported previously among men with atopic diseases, specifically asthma [8], those using anti-asthma medications [21] and those positive for serum allergen-specific IgE, a marker of atopy [7]. However, the effect size in our study was more modest than other studies.

Counter to expectations, we observed a positive association between use of AIMs and risk of prostate cancer, with the strongest effects for non-aspirin-NSAIDs. Given the association was only observed for favourable risk prostate cancer and there was no clear dose response, we again suspect this finding reflects detection bias. While our findings are consistent with those of other large population-based studies (e.g. Skriver et al. 2016 [36], Denmark; Murad et al. 2011, UK [19]), they are inconsistent with meta-analyses which conclude that there is no association [18, 37] or a negative association [20] between prostate cancer and use of non-aspirin-NSAIDs. The meta-analysis by Lui et al. however, did indicate a positive association between non-aspirin-NSAIDs and prostate cancer risk in studies undertaken in Europe. Authors of the Danish population-based study also concluded that the positive association they observed was not likely to be causal [36]. However, findings from studies on the use of non-aspirin-NSAIDs and prostate cancer risk within the context of PSA screening trials do not support effects due to detection bias alone [19, 38].

With respect to inhaled glucocorticoids and prostate cancer risk we did find evidence of a dose-response effect, suggesting there may be a causal relationship. However, it is difficult to disentangle the effects of inhaled glucocorticoids from the effects of asthma, since most prescriptions would be for asthma. There are a number of plausible explanations for increased risk with frequent use of glucocorticoids. Firstly, immunosuppressive effects of glucocorticoids may weaken the body’s defence against nascent or developing tumours [39]. Secondly, metabolic changes induced through sustained use of glucocorticoids (i.e. metabolic syndrome, obesity, hyperglycaemia) have been linked to increased cancer risk [40]. Thirdly, glucocorticoids may directly impact prostate cancer development through ‘cross-talk’ signalling of androgen receptors, leading to upregulation of androgen pathways which stimulate prostate cell growth [41]. However, the lack of dose response with systemic glucocorticoids, and only a weak association with unfavourable risk prostate cancer lessen the argument for a causal relationship. Even if the association between inhaled glucocorticoids and prostate cancer was causal, the effect of prolonged use of these drugs is very modest and would not outweigh the benefits in terms of controlling asthma.

We have used high-quality, registry-based databases to investigate the association between CIDs/AIMs and prostate cancer risk. In the context of autoimmune diseases, which are quite rare, having such a large study population and population-wide coverage is a major strength. Furthermore, we were able to investigate dose-response relationships with respect to duration of CIDs and cumulative dose of AIMs. However, basing our exposure measure on hospital admission data will underestimate the true extent of CIDs since less severe inflammatory diseases (e.g. mild asthma, allergies) are often managed in community or outpatient settings. Likewise, prescription data may also be inaccurate since patients may not complete the course of medication prescribed. In addition we do not have information on prescription-free NSAID use. Furthermore, many AIMs have broad application beyond the management of inflammatory conditions (though this does not nullify their effect on prostate cancer). In terms of timing of initial diagnosis of CIDs, hospital admission data from 1998 may provide too short a time window to completely capture CID history, since conditions may have been diagnosed decades earlier. Even though we examined prostate cancer risk categories and trends according to exposure duration/dose, disentangling the effect of detection bias remains difficult without further information about primary health care visits and frequency of PSA testing.

## Conclusion

This study found no consistent evidence linking prostate cancer risk to history of CIDs or use of AIMs. In general, the increased risk of prostate cancer is likely to be due to detection bias. However, findings suggesting possible associations with asthma/allergies and inhaled glucocorticoids warrant further investigation. More detailed clinical and translational research is required to better understand the relationship between chronic inflammation and prostate cancer risk, given limitations inherent in observational studies.

## Additional files

Additional file 1: **Table S1.** Adjusted Odds for association between AIMs exposure and PCa diagnosis in sensitivity analyses. **Table S2.** Adjusted Odds for association between AIMs exposure and PCa diagnosis in sensitivity analyses (DOCX 17 kb)

Additional file 2: List of conditions and medications included as exposures (DOCX 19 kb)

## Data Availability

The dataset analysed in this study was extracted from the Prostate Cancer Database Sweden (PCBaSe) and are available through the Professor Pär Stattin par.stattin@surgsci.uu.se on reasonable request.

## Abbreviations

AIM: Anti-Inflammatory Medications
ATC: Anatomical Therapeutic Chemical
CCI: Charlson Comorbidity Index
CID: Chronic Inflammatory Disease
ICD: International Classification of Diseases
Ig: Immunoglobulin
LISA: Swedish Longitudinal Integration Database for Health Insurance and Labour Market Studies
NPCR: National Prostate Cancer Register
NPR: National Patient Register
NSAID: Non Steriodal Anti-inflammatory Drug
OR: Odds Ratio
PCBaSe: Prostate Cancer Database Sweden
PSA: Prostate Serum Antigen
STD: Sexually Transmitted disease
