# Supplementary material for: Chronic inflammatory diseases, anti-inflammatory medications and risk of prostate cancer: a population-based case-control study
Source: BMC Cancer. 2019 Jun 21;19:612. doi: 10.1186/s12885-019-5846-3 (PMC6588859; doi:10.1186/s12885-019-5846-3)
Supplement: Supplementary file 1 — Table S1. Adjusted Odds for association between AIMs exposure and PCa diagnosis in sensitivity analyses. Table S2. Adjusted Odds for association between AIMs exposure and PCa diagnosis in sensitivity analyses (DOCX 17 kb) [file 12885_2019_5846_MOESM1_ESM.docx]

**Supplementary Table 1. Adjusted Odds for association between AIMs exposure and PCa diagnosis in sensitivity analyses**

1. AIMs exposure defined as ≥2 prescriptions prior to diagnosis
2. Analysis to PCa diagnoses in 2010 – 2012 only
3. First prescription within 12 months of diagnosis excluded from analysis

| **Cumulative dose of**  **anti-inflammatory medication ^a^** | **AIMs defined as 2+ prescriptions** | **Restricted to PCa Dx 2010-2012** | **1^st^ prescription**  **>12m before PCa Dx** |
| --- | --- | --- | --- |
|  | OR^b^ (95% CI) | OR^b^ (95% CI) | OR^b^ (95% CI) |
|  |  |  |  |
| Any na-NSAID (*reference: no prescriptions*) | 1.27 (1.22-1.30) | 1.23 (1.20-1.26) | 1.29 (1.27-1.32) |
| Acetic acid derivatives | 1.21 (1.16-1.40) | 1.20 (1.16-1.23) | 1.24 (1.21-1.26) |
| Propionic acid derivatives | 1.13 (1.07-1.19) | 1.13 (1.09- 1.17) | 1.12 (1.09-1.25) |
| Cox inhibitors | 1.15 (1.02-1.28) | 1.18 (1.10-1.26) | 1.14 (1.08-1.22) |
| Systemic glucocorticoids | 1.19 (1.14-1.23) | 1.14 (1.10-1.18) | 1.10 (1.07-1.13) |
| Inhaled glucocorticoids | 1.18 (1.13-1.23) | 1.09 (1.03-1.14) | 1.13 (1.08-1.18) |
| Non-steroidal asthma medications | 1.07 (0.96-1.20) | 1.09 (1.03-1.15) | 1.08 (1.04-1.12) |
| Immuno-suppressants | 1.02 (0.94-1.10) | 1.00 (0.91-1.12) | 0.98 (0.91-1.07) |
|  |  |  |  |

**Supplementary Table 2** Interaction between CID and AIM exposure in relation to overall risk of PCa diagnosis

| **Interaction between any CID* and AIMs** | **Interaction between CIDs and AIMS** | |
| --- | --- | --- |
|  | Ratio of ORs (95% CI) | P-value |
| (*reference: no prescription for relevant medication*) |  |  |
| Any na-NSAID | **0.93 (0.87-1.00)** | **0.040** |
| Acetic acid derivatives | **0.91 (0.84-0.97)** | **0.007** |
| Propionic acid derivatives | 1.03 (0.94-1.12) | 0.493 |
| Cox inhibitors | 0.97 (0.80-1.16) | 0.735 |
| Systemic glucocorticoids | **0.86 (0.79-0.94)** | **0.001** |
| Inhaled glucocorticoids* | 0.90 (0.76-1.06) | 0.198 |
| Non-steroidal asthma medications* | 0.99 (0.86-1.15) | 0.930 |
| Immuno-suppressants | 0.97 (0.68-1.37) | 0.874 |
|  |  |  |

*Interaction with asthma/allergies rather than any CID for respiratory glucocorticoids and non-steroidal asthma medications.
