# Supplementary material for: Chronic inflammatory diseases, anti-inflammatory medications and risk of prostate cancer: a population-based case-control study
Source: BMC Cancer. 2019 Jun 21;19:612. doi: 10.1186/s12885-019-5846-3 (PMC6588859; doi:10.1186/s12885-019-5846-3)
Supplement: Supplementary file 2 — List of conditions and medications included as exposures (DOCX 19 kb) [file 12885_2019_5846_MOESM2_ESM.docx]

**LIST OF CONDITIONS AND MEDICATIONS INCLUDED AS EXPOSURES**

**Included inflammatory conditions (ICD 10 Codes):**

**Chronic inflammatory or infectious diseases:** chronic sinusitis (J32), chronic diseases tonsils (J35), chronic laryngitis (J37), chronic bronchitis (J41, J42)**,** emphysema /chronic obstructive pulmonary disease (J43, J44), bronchiectasis (J47)**,** chronic thyroiditis (E06.2, E06.5)**,** gout (M10)**,** osteoarthritis (M15-M19)**,** reactive arthritis (M02.8), other polyarthritis (M13.0), chronic periodontitis (K05.3, K04.5), chronic gingivitis (K05.1), chronic pancreatitis (K86.0, K86.1), Chronic hepatitis (K71.3-K71.9, K72.9, K73, K75.4), chronic viral hepatitis (B18), chronic cholecystitis (K81.1), ulcerative colitis (K25.5-K25.9, K26.4-K26.9, k28.4-k28.9, K29.4-k29.9), chronic nephritis/nephrotic syndrome (N03-N04, N011), chronic cystitis (N30.1-N31.2), pemphigus (L10), diverticulitis (K57), chronic pericarditis (I31.0-I31.1), myositis (M60), chronic osteitis/osteomyelitis (M85.3, M85.3, M86.3-M86.6), vasculitis M31.0, M31.4, M31.5)

**Allergies:** allergic rhinitis (J30, J31)**,** asthma (J45)**,** urticaria (L50)**,** erythema nodosum (L52)**,** atopic dermatitis (L20, L23)**,** eosinophilic esophagitis (K20.0)

**Systemic autoantibody positive autoimmune:** rheumatoid arthritis (M05, M06), systemic lupus erythematosus (M32)**,** dermatomyositis/polymyositis (M33)**,** systemic sclerosis (M34)**,** Sicca/Sjogren’s (M35.0)**,** systemic vasculitis (M35.9).

**Organ specific autoantibody autoimmune:** autoimmune thyroiditis (E06.3), autoimmune hepatitis (K74.4)**,** chronic rheumatic heart disease (I05-I09), Multiple Sclerosis (G35), Addison’s Disease (E27.1), Immune thrombocytopenic purpura (D69.3), Primary biliary cirrhosis (K74.3**,** Discord lupus erythematosus (L93.0)**,** Localised scleroderma (L94.0)**,** myasthenia gravis (G70.0)**,** polyarteritis nodosa (M30)**,** Guillian-Barre Syndrome Polyneuropathy (G61.0), Celiac Disease (K90.0), amyotrophic lateral sclerosis (G12.23)**,** polyangiitis with granulomatosis (M31.3), Raynaud’s disease (I73.0).

**Autoimmune autoantibody negative:** Non-infective Enteritis Colitis (K50 – K52), sarcoidosis (D86), Behcet (M35.2), Polymyalgia rheumatic (M35.3) , Ankylosing spondylitis (M45-M46), enteropathic arthritis (M07), Vitiligo (L80), Psoriasis (L40), Giant cell arteritis (M31.5, M31.6), Reactive Arthritis (M02.3)

**Note:** Chronic prostatitis (N41.1) was excluded from the chronic inflammatory disease group due to concern about the high potential for detection bias.

Type 1 Diabetes (E10) was excluded from the organ specific autoantibody autoimmune disease group due to uncertainty about the accuracy of coding.

**Included anti-inflammatory medications (ATC codes):**

**Non-Aspirin Non-Steroidal Anti-inflammatory Drugs:** (M01A)

Butypyrazolidines(M01AA); acetic acid derivatives (M01AB); Oxicams (M01AC); Propionic acid derivatives (M01AE); Fenamates (M01AG); Coxibs (M01AH); other non-aspirin-NSAIDs (M01AX)

**Immuno-suppressants** (L04A)

**Systemic glucocorticoids** (H02AB)

**Inhaled glucocorticoids** (R03BA)

**Non-steroidal anti-asthma medications:** Short term b-agonists (R03AC02, R03CC03, R03CC53), Long term b-agonists (R03AK06, R03AK07, R03AK08, R03AK10, R03AK11, R03AC12, R03AC13, R03AC18, R03AC19, R03CC12), long acting muscarinic antagonists (R03BB04, R03BB06, R03BB07), Leukotriene receptor antagonists (R03DC03, R03DC01)

**Anti-Gout medications** (M04A)
